# Diversity of small round cell sarcoma in soft tissues around bone among Indian patients

**DOI:** 10.6026/97320630019871

**Published:** 2023-08-31

**Authors:** Rani Singh Guddi, Kumari Bharti, Anuja Mishra, Dinesh Kumar Sinha, Debaditya Haldar, Shabana Azad

**Affiliations:** Department of Pathology, Indira Gandhi Institute of Medical Sciences, Bihar, India; PathologyConsultant Pathologist, Indira Pathlabs (A unit of Indira IVF Pvt. Ltd.), Patna, Bihar, India; Department of Radiation Oncology, Indira Gandhi Institute of Medical Sciences, Bihar, India; Department of Pathology, IGIMS, Patna, Bihar, India; Oncopathology, Homi Bhabha Cancer Hospital, Varanasi, Uttar Pradesh, India

**Keywords:** Small round cell sarcoma, immune histochemistry, tumor

## Abstract

Round cell tumors are a group of malignant tumors which shows overlapping microscopic features of small round monotonous cells with hyper-chromatic nucleus. It mostly occurs in children, adolescent, and young adults. The ancillary technique to confirm the differential diagnosis of round cells sarcoma is immuno-histo chemistry (IHC). Therefore, it is of interest to document the diversity of small round cell sarcoma in soft tissues around bones among Indian patients using IHC. A total of 334 cases among Indians were studied. Among them 160 cases were Non-Hodgkin's Lymphoma, 82 cases are poorly differentiated carcinoma and 92 cases of round cell sarcoma. Out of 92 cases, there were (40%) 27 cases of Wilms tumour, with the highest incidence. The highest incidence was observed in 0-14 years of age group with highest incidence in males. The distribution and diverse histology of different small round cell sarcoma offers challenge in the diagnosis by histopathology. Most frequent round cell tumour is Wilms tumour, followed by Rhabdomyosarcoma. Data shows the role of IHC in classifying soft tissue sarcoma but some time result of IHC remains inconclusive, where cytogenetic is important.

## Background:

Small, uniform, and undifferentiated cells with an inflated nucleus-to-cytoplasm ratio characterize the extremely malignant tumors known as "round cell tumors" [1]. Small, circular, and largely undifferentiated cells are typical of this kind of neoplasm [2]. Differentiating tiny round cell tumors from one another is challenging on histology since they have very similar morphologies. The se tumors affect the skeleton or soft tissues, and are most common in children, adolescents, and young adults [3]. Microscopically, they often lack distinction. Immunohistochemistry (IHC) has been the most often used auxiliary method because it is the most practical adjunct tool to the histopathology that allows for precise decision making [4]. Therefore, it is of interest to document prevalence of small round cell sarcoma of soft tissue and bone. It is also of interest to study the role of IHC in classifying soft tissue sarcoma and differentiating it from poorly differentiated carcinomaandly mphoma.

## Materials and Methods:

## Study Design:

The study was conducted within the pathology division of IGIMS, a tertiary care referral hospital. A retrospective analysis was carried out using a three-year period spanning from January 2018 to December 2021. The study focused on all biopsies of bone and soft tissue that were submitted to the histopathology lab and identified as small round cell tumors. Ethical approval was obtained from the institution's ethical review board. Patient information was sourced from internal files. Histo-morphological details were reevaluated, and Haematoxylin and eosin-stained slides were acquired. Biopsy tissue had been preserved in 10% formalin, embedded in paraffin wax, and stained with Haematoxylin and eosin for histopathological analysis.

## Methodology:

The subsequent analysis involved the examination of immuno-histochemical stained sections to categorize malignant small round cell tumors into sarcomatous soft tissue and bone tumors, as well as non-sarcomatous soft tissue tumors. Additionally, sub-categorization of soft tissue sarcomas was performed. The panel of immuno-histochemical stains was selected based on histo-morphological features and relevant clinical information. Primary IHC Panel including Vimentin, CK, and LCA was employed to categorize tumors into lymphoma, poorly differentiated carcinoma, and small cell sarcoma. Proper controls were implemented for all stains to ensure reliable results. Subsequently, small round cell sarcomas (excluding lymphoma and poorly differentiated carcinoma) were further sub-categorized using a Secondary IHC panel into Wilms tumor (WT1, PAX8, and BCL2), Ewings sarcoma (CD99, FLI1), Rhabdomyosarcoma (Desmin, myogenin, Actin, and SMA), Neuroblastoma (S100, NSE, Synaptophysin, Chromogranin), and synovial sarcoma (TLE1). All stains were performed with appropriate controls to ensure reliable outcomes. Poorly preserved slides and patients lacking sufficient clinical and radiological details were excluded. Externally reviewed soft tissue and bone biopsy slides were not considered, and patients with a history of prior chemotherapy were also excluded.

##  Data analysis:

Statistical analysis was conducted using SPSS version 20.0. Descriptive statistics were used to analyze the data. The prevalence of small round cell sarcoma and the distribution of different subtypes of small round cell tumors were calculated, along with their age and sex distributions.

## Results:

**Figure 1:**
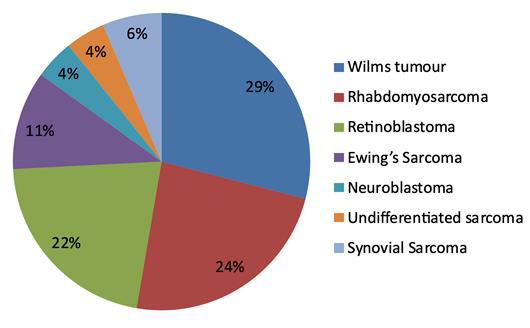
Distribution of different subtypes of small round cell tumors

In the current study encompassing a total of 334 cases, an extensive analysis was conducted based on Histopathological Examination (HPE) and Immunohistochemical (IHC) evaluation in the context of small round cell tumors. The primary IHC panel, comprising Vimentin, CK, and LCA, was utilized to discern distinct pathological entities. Among the cases, those positively identified as poorly differentiated carcinoma (82 cases, 24.55%) and lymphoma (160 cases, 47.90%) was subsequently excluded from the study. This initial screening led to the diagnosis of 92 instances of small round cell sarcoma involving soft tissue/bone after Vimentin positivity. Further classification was undertaken through a secondary IHC panel, refining the study focus to these 92 cases. The prevalence of round cell sarcoma within the tertiary care center was determined to be 27.544%. Subsequently, within this subgroup, the round cell sarcomas were classified into different subtypes: Wilms tumor (27 cases), Rhabdomyosarcoma (22 cases), retinoblastoma (20 cases), Ewing sarcoma (10 cases), undifferentiated sarcoma (3 cases), neuro-blastoma (4 cases), and synovial sarcoma (6 cases) using a combination of histopathological and IHC assessments as indicated in Figure 1. Specifically, Table 1 showcases the Immunohistochemical panel positivity in Wilms tumor, highlighting markers such as WT1 (Wilms tumor 1), PAX8 (Paired-box gene-8), and BCL2 (B-cell lymphoma 2). In a similar vein, Table 2 illustrates the IHC panel positivity in Rhabdomyosarcoma, indicating markers like SMA (Smooth Muscle Actin). (Table 3, Table 4) depicts the IHC panel positivity in Ewing sarcoma, highlighting markers including CD (Cluster of Differentiation), FLI-1 (Friend Leukemia Integration 1), and P-63 (Tumor protein 63). Table4 elucidates the IHC panel positivity in neuro-blastoma. Interestingly, the study findings also include observations on Retinoblastoma, where HPE revealed sheets of small round cells, with instances of true and pseudo rosette formation, as well as necrosis in some cases. Among the 6 synovial sarcoma cases, all exhibited positivity with TLE1 and three cases demonstrated CD99 positivity. Notably, three cases displayed ubiquitous positivity on the secondary IHC panel, yet remained diagnostically challenging, warranting their classification as undifferentiated sarcoma. A predilection towards male predominance was apparent in these tumors, and the majority of cases were observed in children under 14 years of age, with the emergence of small round cell sarcomas typically occurring after the age of 5 years. This study contributes a comprehensive insight into the prevalence and diverse categorization of round cell tumors, underscoring the significance of histopathological and IHC assessments in refining diagnostic accuracy and management strategies.

## Discussion:

The diagnosis of small round cell sarcoma presents a formidable challenge due to the wide spectrum of locations and histologist exhibited by these tumors. Emerging as a diverse group, primary small round cell sarcomas share common histo-morphologic features, immunophenotypes, and clinical attributes, contributing to diagnostic intricacies and the overlapping nature of their characteristics. Within our tertiary care center, the observed prevalence of small round cell sarcoma was documented at 27.544%. Wilms' tumor emerged as the predominant subtype, accounting for 29.34% of cases, closely followed by Rhabdomyosarcoma at 23.92%. Immunohistochemical analyses played a pivotal role in enhancing the understanding of these lesions and assisting in differential diagnoses. The significance of this study lies in its reaffirmation of the utility of immunohistochemistry in subcategorizing soft tissue sarcomas while distinguishing them from poorly differentiated carcinomas and lymphomas. Notably, instances displaying aberrant antigen expression were categorized as undifferentiated round cell sarcomas, necessitating ancillary techniques such as cytogenetics for definitive diagnosis.

In the realm of specific subtypes, the outcomes of this study resonated with previous research in various facets. For instance, among Wilms' tumor cases, an overwhelming 100% displayed positivity with WT1, while 22.22% and 7.40% exhibited positivity with PAX8 and BCL2, respectively, aligning with findings from earlier studies. Similarly, among Retinoblastoma cases, the presence of sheets of small round cells with minimal cytoplasm, as well as the occasional occurrence of true and pseudo rosettes and necrotic debris, echoed the observations of Singh *et al.* [6]. A harmonious match was observed in confirming monophasic Synovial sarcoma cases, guided by the positive TLE-1 criterion, as demonstrated by Wai Chin Foo *et al.* [7]. However, as shown in the work of Sajid H. Shah [5], a subset of cases exhibited overlapping IHC findings, indicative of a diagnostic challenge that may necessitate supplementary approaches such as cytogenetics and ultra-structural examinations to arrive at a definitive conclusion [3].

Diagnosing soft tissue sarcomas is accentuated by their origin in the mesenchyme, and they stand out as an exception among rare cancers. With a higher incidence in children compared to adults, soft tissue sarcomas commonly manifest in extremities. A precise and timely diagnosis significantly influences prognosis and treatment effectiveness for these tumors. Traditional diagnostic methods such as open incisional biopsy, boasting accuracies between 91% and 96%, remain valuable tools. However, the advent of less invasive percutaneous core needle biopsy (CNB), characterized by accuracy rates ranging from 80% to 98%, has further shaped diagnostic strategies, particularly as a preferred initial approach due to its reduced risk of complications. This underscores the continual pursuit of accuracy and minimally invasive techniques to enhance diagnostic efficiency in the field of soft tissue sarcoma assessment. The study's findings were analyzed and compared with other research. Among round cell tumors, non-hodgkin's lymphoma exhibited the highest incidence (26.1%), followed by Ewing's sarcoma/peritoneal carcinomatosis (8.7%) and rhabdomyosarcoma (7%).

These results were consistent with the studies conducted by Patel *et al.* [3] and Shah *et al.* [5]. Patel *et al.* [3] also reported that the median age for patients with Neuroblastoma and Retinoblastoma was 3.5 and 1.5 years, respectively. Regarding age-wise distribution, our study similarly revealed that the pediatric age group (0-14 years) was most affected, aligning with Patel *et al.*'s study [3]. In Ewing's sarcoma, our findings were akin to those of Brahmi *et al.* [8] and Chang TK *et al.* [9], both showing positivity with CD99 (70%) and FLI-1 (100%). In Rhabdomyosarcoma, we observed 100% positivity for desmin and myogenin consistent with Vannunik *et al.* [11] and Rossia *et al.* [12]. Actin displayed 77.2% positivity, slightly lower than Vannunik *et al.*'s study. For neuroblastoma, chromogranin showed 25% positivity, similar to Brahmi *et al.* findings, while NSE exhibited 100% positivity, differing from their 66% positivity.

Out of 27 Wilms tumor cases, 100% displayed positivity with WT1, 22.22% with PAX8, and 7.40% with BCL2. These outcomes aligned with prior studies. Among 20 Retinoblastoma cases, all exhibited sheets of small round cells with scant cytoplasm. Some displayed true and pseudo rosettes, while others contained necrotic debris, a consistent finding with Singh *et al.* [6]. Monophasic Synovial sarcoma was confirmed in only 6 cases, following the criteria of positive TLE-1, in line with Wai Chin Foo *et al.* [7]. Similar to Sajid H. Shah's study [5], our study identified 3% of cases with overlapping IHC findings, while 12.5% had ambiguous results, warranting additional methods like cytogenetics and ultra-structural examinations for final diagnosis [3]. Mesenchyme-originating rare cancers, like soft tissue sarcomas, remain an exception, being more frequent in children than adults and typically manifesting in extremities [13, 14]. A timely and accurate diagnosis can significantly impact prognosis and treatment efficacy for these tumors. While conventional open incisional biopsy boasts accuracy rates of 91% to 96% [13, 14], the less invasive percutaneous core needle biopsy (CNB), with its high accuracy (80% to 98%), is commonly used for initial soft tissue mass biopsies [13, 14,15,16]. This highlights the need for precise diagnostic techniques, given the potential complications associated with traditional methods.

## Conclusion:

The prevalence of small round cell sarcoma is 27.544%, with Wilms' tumor being the most prevalent subtype (29.34%), followed by Rhabdomyosarcoma (23.92%). Immuno-histochemical analyses serve as valuable tools in characterizing these lesions and aiding in differential diagnosis.

## Figures and Tables

**Table 1:** Shows IHC Panel positivity in Wilms tumor WT1- Wi1m's tumor 1, PAX8- Paired-box gene-8, BCL2-B- cell lymphoma 2

**No. of cases**	**WT1**	**PAX8**	**BCL2**	**% age of positivity**
19	++	--	--	70.37%
6	++	+	--	22.22%
2	++	--	+	7.40%

**Table 2:** Shows IHC Panel positivity in Rhabdomyosarcoma SMA- Smooth Muscle Actin

**No. of cases**	**SMA**	**Desmin**	**Myogenin**	**Actin**	**% of positivity**
8	+	++	+	+	36.36%
9	++	++	++	+	40.90%
5	--	++	++	--	22.72%

**Table 3:** Shows IHC Panel positivity in Ewing sarcoma CD- Cluster of differentiation, FLI-1- Friend Leukemia integration1, P-63- Tumor protein 63

**No. of cases**	**CD99**	**FLI-1**	**P-63**	**% age of positivity**
7	++	++	--	70%
3	--	++	+	30%

**Table 4:** Shows IHC Panel positivity in neuro-blastoma

**No. of cases**	**Neuron specific enolase**	**Chromogranin**	**Synaptophysin**	**% age of positivity**
3	++	--	--	75%
1	++	+	+	25%
